# Neurothrombectomy for Acute Ischemic Stroke Across Clinical Trial Design and Technique: A Single Center Pooled Analysis

**DOI:** 10.3389/fneur.2020.01047

**Published:** 2020-09-24

**Authors:** Tudor G. Jovin, Shashvat M. Desai, Amin Aghaebrahim, Andrew F. Ducruet, Dan-Victor Giurgiutiu, Bradley A. Gross, Maxim Hammer, Brian T. Jankowitz, Mouhammad A. Jumaa, Cynthia Kenmuir, Guillermo Linares, Vivek Reddy, Marcelo Rocha, Matthew Starr, Viktoria Totoraitis, Lawrence Wechsler, Syed Zaidi, Ashutosh P. Jadhav

**Affiliations:** ^1^Department of Neurology, University of Pittsburgh Medical Center, Pittsburgh, PA, United States; ^2^Department of Neurosurgery, University of Pittsburgh Medical Center, Pittsburgh, PA, United States

**Keywords:** stroke, thrombectomy, ischemic stroke, clinical trial, technique

## Abstract

**Introduction:** The practice of endovascular therapy has evolved dramatically over the last 10 years with randomized clinical trials investigating the benefit of thrombectomy in select patient populations based on time of presentation, imaging criteria, and procedural technique. We sought to understand the benefit of thrombectomy in patients treated within the context of a clinical trial at a single academic center.

**Methods:** Patient-level data recorded in case forms and core-lab adjudicated data were analyzed from patients enrolled in RCTs investigating the benefit of endovascular thrombectomy over medical management (IMSIII, MR RESCUE, ESCAPE, SWIFT PRIME, and DAWN) between 2007 and 2017 at a single academic referral center.

**Results:** A total of 134 patients (intervention group, *n* = 81; medical group, *n* = 53) were identified across five clinical trials (IMSIII, *n* = 46; MR RESCUE, *n* = 4; ESCAPE, *n* = 24; SWIFT PRIME, *n* = 14; DAWN, *n* = 46). There were no significant differences between the treatment arm and control arm in terms of age, gender, baseline NIHSS, ASPECTS, and site of occlusion. Rates of good outcome were superior in the intervention group with early neurological recovery (NIHSS of 0–1 or increase NIHSS of 8 points at 24 h) at a higher rate of 49% vs. 17% (*p* = <0.001) and higher rates of functional independence (90 day mRS 0–2 of 53% vs. 26%, *p* = 0.002). In multivariate logistic regression analysis, lower NIHSS and younger age were predictors of good outcome. There were comparable rates of good outcome irrespective of clinical trial, imaging selection criteria (CTP vs. MRI), early vs. late time window (0–6 h vs. 6–24 h) and procedural technique (Merci vs. Solitaire/Trevo device). There were no differences in rates of sICH, PH-2 or mortality in the intervention group vs. medical group.

**Conclusions:** At a large academic center, the benefit of endovascular therapy over medical therapy is observed irrespective of clinical trial design, patient selection or procedural technique.

## Introduction

Acute ischemic stroke due to large vessel occlusion (LVO) is a devastating disease with a dismal natural history (1, 2). For more than two decades, intravenous thrombolysis using tissue plasminogen activator was the only FDA approved treatment for disability reduction—with strict indications for use and limited efficacy (3). Large randomized clinical trials have explored the safety and efficacy of endovascular thrombectomy for the treatment of acute ischemic stroke due to LVO, to rapidly and effectively achieve reperfusion and salvage ischemic brain tissue (4–11). While the safety of ET was established, many thrombectomy trials failed to prove the superiority of ET over medical management. Reasons for not observing a signal for efficacy could be—improper patient selection [including patients without confirmed intracranial vessel occlusion (4, 12), infarct core imaging, collateral status (9), and/or patients with mild symptoms], delayed treatment (13), and/or use of high risk pharmacological intra-arterial reperfusion therapies or early first generation neurothrombectomy devices (4). Experience with endovascular thrombectomy at high volume centers provides more streamlined systems of care (faster door to groin puncture times) and is associated with higher recanalization rates (14).

Our comprehensive stroke center participated in multiple randomized clinical trials exploring the benefit of endovascular thrombectomy over best medical management for treatment of acute ischemic stroke due to LVO. These trials were different from each other in terms of inclusion criteria: stroke severity, baseline functional status, time since symptoms onset and vessel occlusion. Trials also varied with respect to baseline imaging requirements, reperfusion techniques and devices as well as follow-up protocols and outcome measurements.

We sought to conduct a comprehensive pooled analysis of patients enrolled in the randomized controlled trials at our center and compare intervention arm patients with the control arm and understand a single academic center experience with endovascular thrombectomy over a 10 year period.

## Methods

### Study Inclusion and Procedures

The University of Pittsburgh Medical Center participated in multiple randomized controlled trials investigating new therapies for acute ischemic stroke. We identified five clinical trials (IMS III, MR RESCUE, ESCAPE, SWIFT PRIME, and DAWN) (4, 6, 9, 10, 15) which included patients with acute anterior circulation ischemic stroke and randomized patients to investigate endovascular thrombectomy plus medical management vs. medical management alone. We performed a pooled analysis of these trials to compare the intervention and control arm patients across trial design: patient selection criteria (stroke severity, occlusion location, baseline functional status), time window of treatment, technique and device used to achieve reperfusion.

While IMS III, MR RESCUE, ESCAPE, SWIFT PRIME Trials enrolled patients in the early time window (within 6 to 12 h from stroke onset), the DAWN trial included patients presenting 6–24 h after last known well. With the exception of IMS III and MR RESCUE, all trials demonstrated superiority of endovascular thrombectomy over medical management. Detailed inclusion and exclusion criteria for each trial have been previously published.

After Institutional Review Board approval, we extracted patient level data (demographic and clinical) from trial related subject folders and case-forms. Imaging core lab data were collected, pooled together and analyzed for all of the individual trials, except MR RESCUE (we used center level data for those 4 patients).

### Outcomes

The pre-specified primary efficacy outcome in this pooled analysis was the degree of functional disability on the modified Rankin Scale (mRS) at 90 days. A good functional outcome was defined as mRS score 0–2 and a poor functional outcome was an mRS of more than 2. Secondary efficacy outcome is early neurological recovery as defined as: NIHSS score at 24 h of 0–1 or a decrease in NIHSS score from baseline to 24 h of 8 or more. Pre-specified safety outcomes were the proportion of patients with symptomatic intracranial hemorrhage (per NINDS criteria) (3), parenchymal hematoma type 2 (blood clot occupying>30% of the infarcted territory with substantial mass effect) within 5 days on computed tomography (CT) or magnetic resonance imaging (MRI), and mortality within 90 days. Technical efficacy was based on the degree of revascularization at the end of the endovascular therapy, defined using the modified Thrombolysis in Cerebral Infarction (mTICI) scale score of 2b or more.

### Statistical Analysis

Continuous variables are reported as mean ± SD or median with inter quartile range (as appropriate) and categorical variables are reported as proportions. Between groups comparison for continuous variables was performed using the Student *t*-test and categorical variables using Chi-square test or Fisher Exact Test, as appropriate. Univariate analysis and multivariable logistic regression analysis was performed to identify predictors and adjust for known confounders. The trials differed with respect to definitions of various parameters (such as of time to presentation or time since last seen well), hence we used in-hospital data to standardize the process and definitions. Sub-group analyses were performed and OR with 95% CI have been reported in tabular form and graphically presented as a forest plot. Significance was defined as p <0.05. Statistical analysis was performed using IBM SPSS Statistics 23 (IBM-Armonk, NY).

## Results

### Baseline Characteristics (Table 1)

A total of 134 patients (intervention arm, *n* = 81; control, *n* = 53) were identified across the five clinical trials (IMSIII, *n* = 46; MR RESCUE, *n* = 4; ESCAPE, *n* = 24; SWIFT PRIME, *n* = 14; DAWN, *n* = 46). There were no significant differences between the intervention arm and control arm in terms of age, gender, baseline NIHSS, baseline ASPECTS and site of occlusion.

**Table 1:** Baseline Characteristics.

	**Intervention Arm *n* = 81**	**Control Arm *n* = 53**	***P*-value**
**Demographics**			
Median Age	72 (65–80)	68 (57–80)	0.289
Males	34 (42%)	28 (53%)	0.217
**Past medical history**			
Hypertension	57 (70%)	36 (68%)	0.763
Diabetes Mellitus	16 (20%)	13 (26%)	0.516
Atrial Fibrillation	30 (38%)	11 (22%)	0.081
Tobacco	11 (14%)	6 (11%)	0.700
Past Stroke	10 (13%)	8 (16%)	0.608
Coronary Artery Disease	19 (23%)	14 (26%)	0.697
Congestive heart Failure	8 (10%)	4 (8%)	0.766
**Clinical characteristics**			
Admit NIHSS Score	16 ± 4	16 ± 5	0.231
**Imaging characteristics**			
Baseline ASPECTS	8 (6–10)	9 (6–10)	0.502
**Site of occlusion**			
ICA and ICAT	16 (20%)	7 (13%)	0.325
M1 MCA	56 (69%)	42 (79%)	0.196
M2 MCA	5 (6%)	2 (4%)	0.706
Extracranial IC	0 (0%)	1 (2%)	0.384
No Occlusion/Spontaneous recanalization	4 (5%)	1 (2%)	0.648
**Clinical trial**			
IMS III	32 (40%)	14 (26%)	Randomized trial
MR RESCUE	1 (1%)	3 (5%)	Randomized trial
ESCAPE	14 (17%)	10 (19%)	Randomized trial
SWIFT PRIME	8 (10%)	6 (11%)	Randomized trial
DAWN	26 (32%)	20 (38%)	Randomized trial
**Treatment specifics**			
IV-tPA	45 (56%)	26 (49%)	0.461

### Outcomes (Table 2; Figure 1)

Rates of good outcome were superior in the intervention group with early neurological recovery (defined as NIHSS of 0–1 or decrease in NIHSS of ≥8 points at 24 h) at a rate of 52% vs. 13% (*p* = <0.0001) as well as higher rates of functional independence (90 day mRS 0–2 of 53% vs. 26%, *p* = 0.002). In multivariate logistic regression analysis, lower baseline NIHSS (*p* = 0.003) and younger age (*p* = 0.03) were independent predictors of good outcome. There were no differences in rates of sICH, PH-2 or mortality in the intervention arm vs. medical arm. Of the 81 patients in the intervention arm, 91% (*n* = 74) achieved successful reperfusion (mTICI score ≥ 2b).

**Table 2:** Efficacy and Safety Outcomes.

		**Intervention Arm *n* = 81**	**Control Arm *n* = 53**	***P*-value**
mRS at 90 d	mRS 0–2	43 (53%)	14 (26%)	0.002
	mRS 3–6	38	39	
NIHSS score at 24 h	0–2	15 (19%)	3 (6%)	0.032
	>2	66	50	
Early neurological recovery at 24 h	Yes	42 (52%)	7 (13%)	<0.0001
	No	39	46	
Symptomatic intracranial hemorrhage		2 (2.4%)	0 (0%)	0.518
Parenchymal Hematoma Type 2		4 (4.9%)	1 (2%)	0.362
Mortality		16 (20%)	9 (17%)	0.687

**Figure 1:**
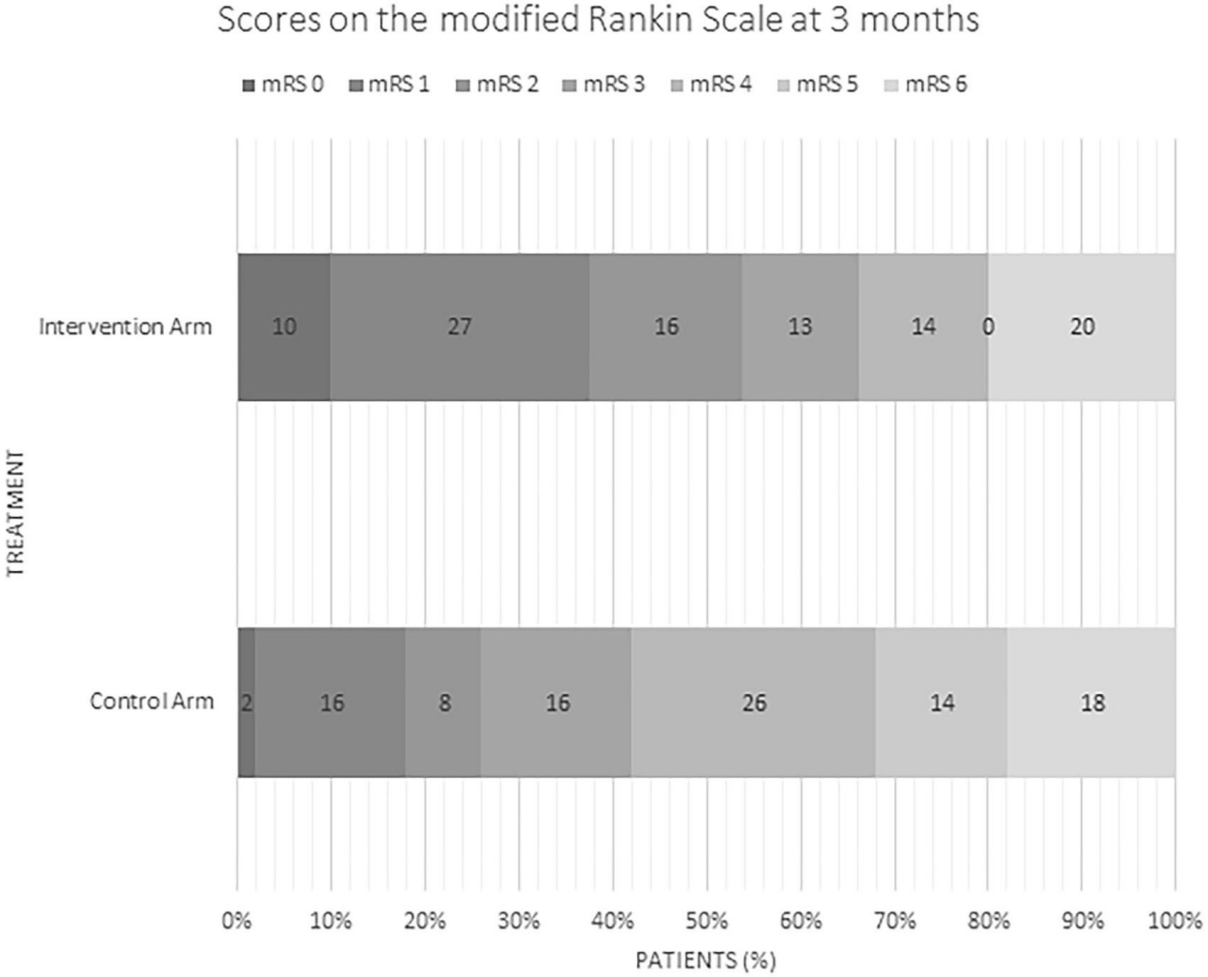
Distribution of mRS scores in intervention and control arm.

### Outcome Comparison by Trial and Different Subgroups

#### By Trial

**Figure 2:**
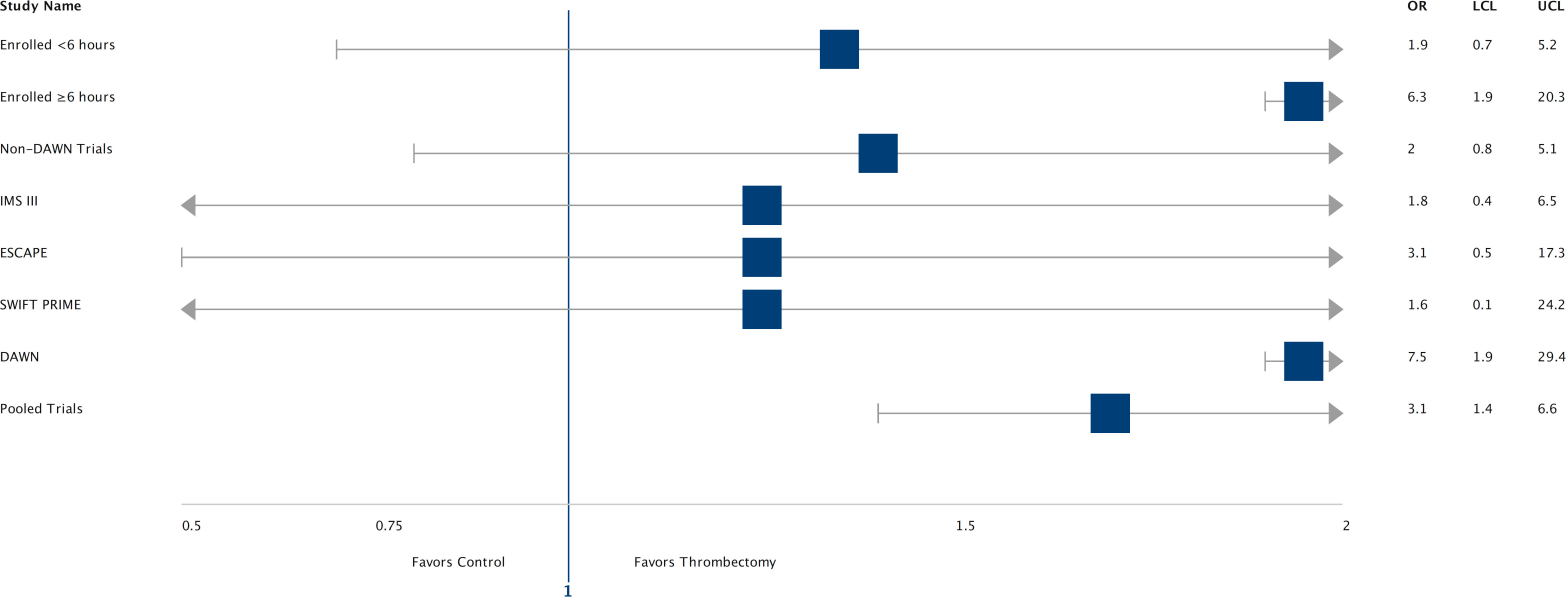
Forest plot: Trials. OR, Odds ratio; UCL and LCL, upper and lower confidence limit.

Pooled data from all five trials showed reduced chance of disability at 90 days in the intervention arm patients vs. control arm patients (OR-3.1, CI- 1.4–6.6). The odds ratio and confidence intervals for individual trials, subjects enrolled <6 h and >6 h, and non-DAWN trials have been described in Figure 2. Rates of successful reperfusion by trial were the following: 88% (28) in IMS III (total = 32), 100% (1) in MR RESCUE (total = 1), 100% (8) in SWIFT PRIME (total = 8), 93% (13) in ESCAPE (total = 14), and 92% (24) in DAWN (total = 26).

#### By Sub-groups

**Figure 3:**
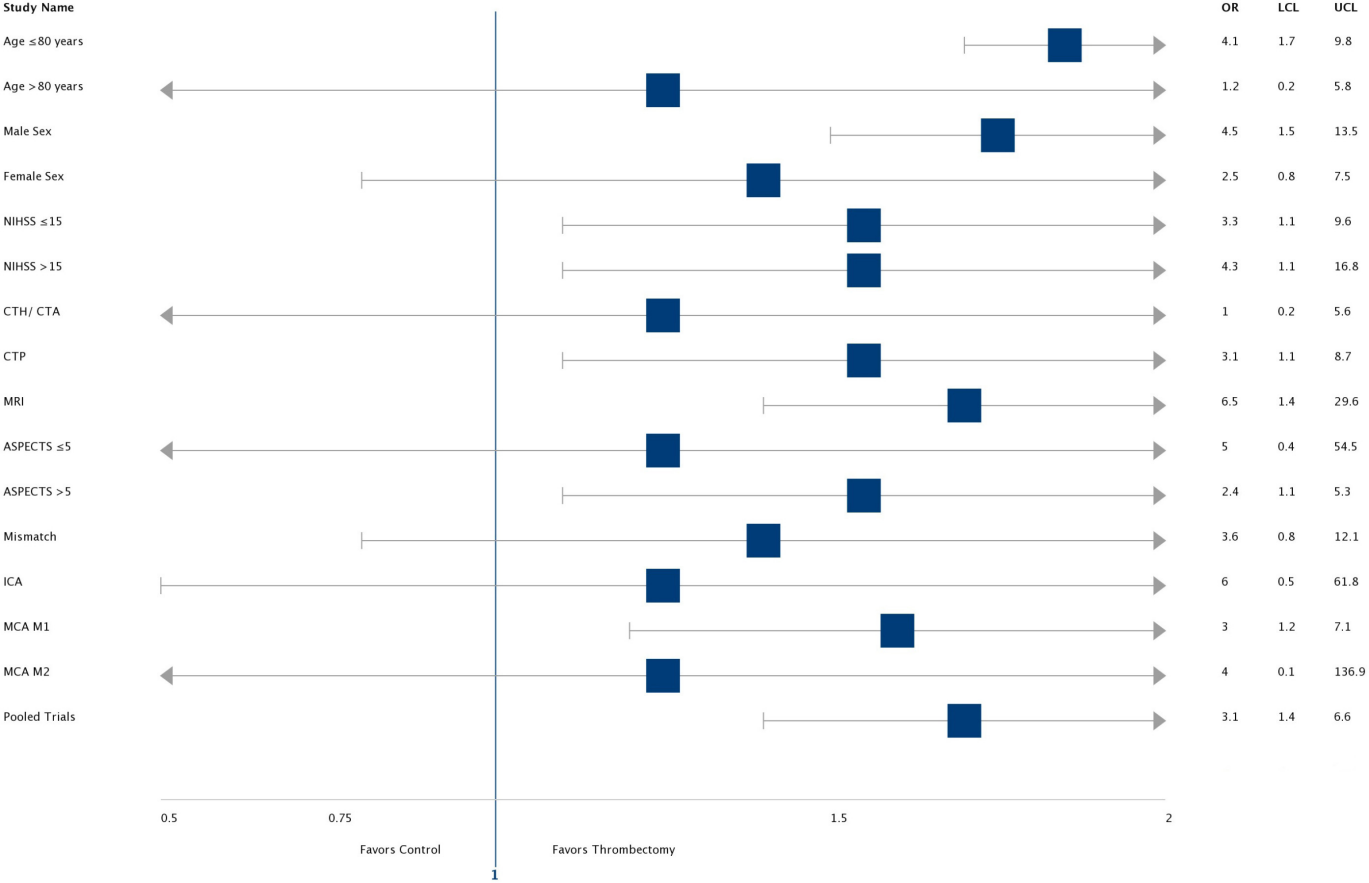
Forest Plot: Sub-groups. OR, Odds ratio; UCL and LCL, upper and lower confidence limit.

The odds ratio and confidence interval for reducing disability at 90 days by endovascular thrombectomy when compared to medical management in various sub-groups are demonstrated in a forest plot (Figure 3). Most subgroups (including high NIHSS score patients) favor intervention with ET. In patients with age over 80 years and ASPECTS < 5, the CI crosses 1. Selection with advanced imaging (OR 6.5, CI- 1.4–29.6) led to higher odds of a good outcome. In the intervention arm, there were higher rates of good outcomes in patients who were successfully reperfused (TICI ≥ 2B group: 58% vs. in TICI <2B group-20%, *p* = 0.09). However, rates of good outcomes and reperfusion were comparable, irrespective of imaging selection criteria (CTA vs. CTP vs. MRI = 50% vs. 48% vs. 67%, *p* = 0.31) and procedural technique (Merci vs. Solitaire/Trevo = 45% vs. 55%, *p* = 0.45). In the control arm patients, rates of good outcomes were comparable between patients who received IV tPA and those who did not (IV tPA vs. non-IV tPA: 32% vs. 20%, *p* = 0.33).

The predictive value of early NIHSS score changes on 90 day outcome was assessed in patients who underwent ET. Three distinct subgroups were identified—Group 1 (same as early neurological recovery group as defined previously), Group 2 (modest neurological recovery- drop of NIHSS score between baseline and at 24 h of 0–7), Group 3 (NIHSS decay- increase in NIHSS score between baseline and at 24 h). Good outcome rates were statistically different (*p* < 0.001) in these groups—Group 1 (69%), Group 2 (50%), and Group 3 (8.3%).

### Comparisons

Rates of mRS 0–2 at 90 days (53% vs. 46%, *p* = 0.23), symptomatic intracranial hemorrhage (2.4% vs. 4.4%, *p* = 0.41) and mortality (20% vs. 15.3%, *p* = 0.30) were comparable between data from our pooled analysis and the HERMES metanalysis.

For IMS III intervention arm patients, rates of successful recanalization (TICI ≥ 2b) (90% vs. 43%, *p* = <0.001) and time from start of IV tPA administration to groin puncture (54 ± 28 vs. 81 ±27 min, *p* = < 0.001) were significantly better for IMS III patients enrolled at our center compared to overall trial results.

## Discussion

The results of our pooled analysis of 134 randomized patients between five trials are consistent with the results of major randomized controlled trials and the HERMES meta-analysis (2). Overall, ET is significantly superior to medical management in our pooled analysis with differences (between intervention and control arm patients) in rates of early neurological improvement of 39% and rates of mRS 0–2 at 3 months of 27%. The overall number needed to treat (NNT) is 3.7. ET is safe, and rates of hemorrhage and mortality are comparable in the two arms.

Pooling individual patient level data at a single center offered several advantages. We enrolled ~10% of the total patients enrolled in these trials. We were able to standardize efficacy and safety outcome definitions and access blinded assessments of neuroimaging from core laboratories of the trials. The five trials, exclusive in design, were conducted over different eras of evolution of ET using varied selection criteria, different time window of enrollment and used a broad range of devices and techniques, offering insight into different subgroups of interest.

Younger age, low baseline NIHSS score, favorable ASPECTS and successful recanalization are important predictors of achieving a good functional outcome. We find persistent treatment effect of ET across most predetermined subgroups. Patients over 80 years of age benefitted from ET in the DAWN trial (likely due to better selection) and the ESCAPE Trial (which focused on efficient workflow). Higher baseline infarct burden patients (ASPECTS < 5) may also benefit from ET if recanalization is achieved. Likely explanations include lower rates of decompressive hemi-craniotomy, higher disability at 3 months and/ or mortality. LVO strokes with mild NIHSS score also benefitted from ET although the sample size is too small in our study to address this population. Rates of good outcome at 90 days were higher in patients who were successfully reperfused (TICI ≥ 2B group: 58.3% vs. in TICI < 2B group: 20%). Comparable successful reperfusion rates (*p* = 0.33) were observed between MERCI (87.5%) and Solitaire/Trevo (93.75%). Specific technique and/ or device utilized is likely irrelevant if successful reperfusion (mTICI 2b or more) is achieved.

In our cohort, 78% (105) patients are eligible for thrombectomy per 2018 American Heart Association Guidelines (16), with ASPECTS <6 (6%) and distal occlusion (6%) or absence of occlusion (4%) being the most common reasons for ineligibility. The trials required patients to meet strict selection criteria to be eligible for enrollment. A simpler and broader selection paradigm would expand indications for ET.

Prior to publication of the five landmark trials in 2015, the future of ET as the mainstay of treatment of AIS due to LVO was in doubt. IMS III trial enrolled over 650 patients and rendered a neutral result. Two pertinent questions arise: (1) 35% of patients in our pooled analysis are IMS III patients and yet we see strong benefit of thrombectomy. (2) Why did the landmark trials in 2015 succeed? Sub-group analyses and secondary results from IMS III trial indicated that there was a strong trend toward benefit of thrombectomy in patients with radiographically confirmed large vessel occlusion (12) (a key feature of the HERMES trials) and who achieved high-quality (mTICI 2b or (3) recanalization. In the IMS III trial, patients with ICA/ M1 occlusion undergoing thrombectomy, achieved 42.5% rate of TICI2b/3 flow as compared to the IMS III intervention arm patients enrolled at our center (90%) and the HERMES meta-analysis intervention arm patients (71%) (2, 12). Further, time from IV tPA to groin puncture in the IMS III trial was 81 ± 27 min compared to 54 ± 28 min for IMS III patients at our center (*p* = < 0.001) (13). Speed and quality of recanalization were significantly better for IMS III patients in our pooled analysis compared to the overall IMS III results. Technical expertise and efficacious workflow systems at a large high-volume center are likely important contributors to realize the full potential of thrombectomy. Additionally, care of the post-thrombectomy patient is vital to the success of thrombectomy (17).

The treatment effect is highest in the DAWN trial due to precise physiology-based patient selection and the poor natural history of the control arm. Imaging and physiology-based patient selection will be important to treat patients with ET, especially in late time windows (10). There is a trend toward better outcomes in patients randomized in the late time window. This is paradoxical given that “time is brain.” The NNT for patients randomized before 6 h is 7.7 whereas the NNT for patients randomized after 6 h is 2.2. This apparent inconsistency can be explained by superior outcomes in the treatment arm due to better patient selection and inferior outcomes in the control arm mostly due to lack of administration of tissue plasminogen activator.

Optimizing imaging criteria into systems of care will be important to improve patient selection and outcomes. Identifying an intracranial LVO responsible for ischemic stroke symptoms using CT angiography or MR angiography at a referring facility can direct patient triage to an ET capable center. Patients selected by advanced imaging modalities like CT perfusion or MRI tend to have higher rates of favorable outcome, due to accurate delineation of ischemic core and/or penumbra.

A strength of this study is that it reports on the experience of a large single center analysis of randomized data (including core lab assessed neuroimaging) over a crucial period of evolution of endovascular therapy. The importance of consecutive enrollment cannot be understated. Our center had a policy to offer thrombectomy to trial-eligible patients only in the context of a trial, thus minimizing patient selection bias. This study reinforces the need to increase the level of expertise of neuro-interventionalists and to establish adequate workflow protocols to reduce time from first medical contact to reperfusion of LVO strokes. Attempts to abandon formal training and experience standards for physicians performing neurothrombectomy should be discouraged (18) as this risks offering thrombectomy at inexperienced centers with potentially suboptimal results.

## Limitations

First, the selection of trials is prone to bias. We chose all anterior circulation ischemic stroke trials and the number of trials is relatively small. Second, the trials differed in patient selection and treatment effects, leading to various factors (like patient volume per trial) driving overall treatment effect, despite individual patient data providing greater flexibility. Third, a sample size of 134 may be under-powering the study in certain subgroup analyses.

## Conclusion

Our data demonstrates the safety, technical feasibility and efficacy of endovascular therapy in different subgroups of acute ischemic stroke patients who harbor an LVO, selected using different clinical and imaging selection paradigms, across time windows of treatment using various techniques and devices to achieve reperfusion.

## Data Availability Statement

The original contributions presented in the study are included in the article/supplementary material, further inquiries can be directed to the corresponding author/s.

## Ethics Statement

The studies involving human participants were reviewed and approved by Institutional Review Board of University of Pittsburgh. The patients/participants provided their written informed consent to participate in this study.

## Author Contributions

AJ and TJ: conception and design, study supervision, and contributed equally to the manuscript development and preparation. AJ and SD: drafting the article. All authors: acquisition of data, analysis and interpretation of data, critically revising the article, and administrative/technical/ material support.

## Conflict of Interest

TJ - Consultant: Neuravi, CodmanNeurovascular, StrykerNeurovascular, Fundacio Ictus. Stock: Anaconda, Silk Road, Blockade Medical. AD - Consultant: Penumbra, Stryker, Medtronic, Cerenovus, and Koswire. BG - Consultant: Microvention, Medtronic. The remaining authors declare that the research was conducted in the absence of any commercial or financial relationships that could be construed as a potential conflict of interest.

## Abbreviations

AIS: acute ischemic stroke
ASPECTS: Alberta stroke program early CT score
CT: computed tomography
ICA: internal carotid artery
LVO: large vessel occlusion
MCA: middle cerebral artery
NIHSS: National Institutes of Health Stroke Scale
sICH: symptomatic intracerebral hemorrhage.
